# OSU-T315 as an Interesting Lead Molecule for Novel B Cell-Specific Therapeutics

**DOI:** 10.1155/2018/2505818

**Published:** 2018-09-12

**Authors:** Kristien Van Belle, Jean Herman, Mark Waer, Ben Sprangers, Thierry Louat

**Affiliations:** ^1^Interface Valorisation Platform (IVAP), KU Leuven, 3000 Leuven, Belgium; ^2^Laboratory of Experimental Transplantation, Department of Microbiology & Immunology, KU Leuven, 3000 Leuven, Belgium; ^3^Department of Pediatric Nephrology and Solid Organ Transplantation, University Hospitals Leuven, 3000 Leuven, Belgium; ^4^Department of Nephrology, University Hospitals Leuven, 3000 Leuven, Belgium

## Abstract

B cells are pathogenic in various disease processes and therefore represent an interesting target for the development of novel immunosuppressants. In the search for new therapeutic molecules, we utilized an *in vitro* B cell activation assay with ODN2006-stimulated Namalwa cells to screen a chemical library of small molecules for B cell modulating effects. OSU-T315, described as an inhibitor of integrin-linked kinase (ILK), was hereby identified as a hit. On human and murine primary B cells, OSU-T315 potently suppressed the proliferation and the production of antibodies and cytokines upon stimulation, suggesting that ILK could be a promising target in the modulation of B cell activity. Mice with B cell-specific knockout of ILK were generated. Surprisingly, knockout of ILK in murine B cells did not affect B cell function as assessed by several *in vivo* and *ex vivo* B cell assays and did not alter the B cell immunosuppressive activity of OSU-T315. In conclusion, OSU-T315 displays potency as B cell modulator, probably through a mechanism of action independent of ILK, and might serve as lead drug molecule for the development of novel B cell-selective drugs.

## 1. Introduction

At the present time, there are few B cell-specific immunomodulatory agents available and applicable for clinical purposes and they usually aim for a depletion of B cell population(s). These include monoclonal antibodies directed against B cell surface markers, such as rituximab, ocrelizumab, epratuzumab, or directed against B cell growth factors, such as belimumab, and small molecule agents like Bruton's tyrosine kinase (BTK) inhibitor ibrutinib and the proteasome inhibitor bortezomib. Hence, there is an unmet need for new B cell drugs that aim for a modulation of B cell's activation status. Recently, we described the oligodeoxynucleotide (ODN) 2006-stimulated Namalwa cell line as a relevant, homogeneous, and stable B cell activation model by which new targets and inhibitors of the B cell activation processes can be identified through flow cytometric analysis of the expression of activation and costimulatory cell surface markers [1]. In search of innovative B cell immunomodulating agents, this assay was chosen to screen a library of chemical agents for inhibitory effects on activated human B cells. The screening allowed us to identify OSU-T315 as a potentially interesting agent to interfere with human B cell activation. This compound is described as targeting ILK with IC_50_ of 600 nM in an *in vitro* radiometric kinase assay [2]. In previous studies, some murine models with targeted deletion of ILK have been generated to investigate the role of ILK in the different cell populations [3–10]. To our knowledge, ILK has not yet been studied for its role in B cell biology which encouraged us to explore ILK's potential as target for B cell therapeutics by generating mice with B cell-specific genetic deletion of ILK.

## 2. Materials and Methods

### 2.1. Cells and Cell Lines

Human B cell line Namalwa (European Collection of Cell Cultures, ECACC, England) was maintained in culture flasks (TPP, Switzerland) as suspension culture in complete RPMI 1640 culture medium at 37°C and 5% CO_2_. Blood samples of healthy volunteers were collected at the Red Cross of Mechelen, Belgium. Each donor consents to the use of his blood for research purposes. Human peripheral blood mononuclear cells (PBMCs) were obtained by density gradient centrifugation of the heparinized venous blood over Lymphoprep™ (Axis Shield PoC AS; density 1.077 ± 0.001 g/ml). Highly purified naive peripheral human B cells were separated from fresh human PBMCs using magnetic columns by positive selection using cluster of differentiation (CD) 19 magnetic beads according to the manufacturer's instructions (MACS Miltenyi Biotech, Leiden, The Netherlands). The purity of the isolated primary B cells was ≥95% as analyzed by flow cytometry. Cells were suspended at the desired concentration in complete Dulbecco's modified Eagle's medium (DMEM) culture medium. Single-cell suspensions of murine splenocytes were prepared by manual disruption of total spleens, and highly purified B lymphocytes were isolated by immunomagnetic positive selection according to the manufacturer's instructions (STEMCELL Technologies, EasySep™ Mouse CD19 positive selection kit II, Grenoble, France). The purity of the isolated murine B cells was ≥95% as analyzed by flow cytometry. Cells were suspended at the desired concentration in complete DMEM culture medium.

Complete RPMI 1640 culture medium consisted of RPMI 1640 with 10% foetal calf serum (FCS, HyClone® Thermo Scientific, United Kingdom) and 5 *μ*g/ml gentamicin sulphate. Complete DMEM culture medium consisted of DMEM with 10% heat-inactivated FCS and 5 *μ*g/ml gentamicin sulphate. Cell culture media and gentamicin sulphate were purchased from BioWhittaker® Lonza (Verviers, Belgium).

### 2.2. Characterization of OSU-T315 via Functional *In Vitro* Assays with Human Cells

OSU-T315 was purchased from Calbiochem, Merck Millipore (Overijse, Belgium). The measurement of cytotoxicity of OSU-T315 was done on cells of the Namalwa cell line by WST-1 viability assay. The cell proliferation agent WST-1 was purchased from Roche Diagnostics (Mannheim, Germany). OSU-T315 was added at different concentrations to the Namalwa cells. After 48 hours of incubation at 37 °C and 5% CO_2_, Triton® X-100 (0.5% final; Fluka Biochemika, Buchs, Switzerland) was added in control wells. WST-1 reagent was added, and Namalwa cells were incubated for 2 to 4 hours at 37 °C and 5% CO_2_. The absorbance of the formazan dye was measured by the EnVision™ 2103 Multilabel Reader (Perkin Elmer, Zaventem, Belgium) at 540 nM.

An initial evaluation of B cell modulatory effects was done on Namalwa cells and on human primary B cells by flow cytometric analysis on the expression of activation and costimulatory cell surface markers after 24 hours of stimulation with 0.1 *μ*M ODN2006 in presence of compound OSU-T315. Furthermore, OSU-T315 was investigated for its effects on antibody and interleukin (IL) 6 production and B cell proliferation upon activation of human B cells by different stimulatory conditions (0.1 *μ*M ODN2006, Invivogen, Toulouse, France; 1 *μ*M resiquimod, Sigma-Aldrich, Diegem, Belgium; 91 *μ*g/ml TNP-BSA, Biosearch Technologies, Novato, California, USA). For assessment of IL6 production, freshly isolated human CD19^+^ B cells were plated at 50000 cells per well in 220 *μ*l complete DMEM medium and activated by different stimulatory conditions. After 2 days of incubation, supernatant was taken for analysis of IL6 with AlphaLISA human IL6 kit according to the manufacturer's instructions (Perkin Elmer, Zaventem, Belgium). Detection was performed with the EnVision™ 2103 Multilabel Reader (Perkin Elmer, Zaventem, Belgium). For assessment of cell proliferation, freshly isolated human CD19^+^ B cells were plated at 50000 cells per well in 220 *μ*l complete DMEM medium and activated by different stimulatory conditions. 10 *μ*Ci ^3^H thymidine (Perkin Elmer, Zaventem, Belgium) was added in the wells for the last 18 hours of 3 days of incubation. The cells were harvested on glass filter paper (Perkin Elmer, Zaventem, Belgium). After drying, radioactivity was counted in a scintillation counter (TopCount, Perkin Elmer, Zaventem, Belgium). For assessment of immunoglobulin (Ig) production, freshly isolated human CD19^+^ B cells were plated at 25000 cells per well in a 384-well plate (Perkin Elmer, Zaventem, Belgium) in 55 *μ*l complete DMEM medium and activated by different stimulatory conditions. After 7 days of incubation, supernatant was taken for analysis of IgM and IgG with AlphaLISA human IgM and IgG kit according to the manufacturer's instructions (Perkin Elmer, Zaventem, Belgium). Detection was performed with the EnVision™ 2103 Multilabel Reader (Perkin Elmer, Zaventem, Belgium). For human one-way mixed lymphocyte reaction (MLR), freshly isolated human PBMCs were the responder cells and growth-inhibited RPMI 1788 cells served as stimulator cells. To block their proliferation RPMI, 1788 cells were treated with 96 nM mitomycin C (mitomycin C Kyowa®, Takeda Belgium, Brussels, Belgium) for 20 minutes at 37 °C. After three washes with RPMI 1640 medium containing antibiotics, the stimulator cells were then diluted at the desired cell concentration in complete RPMI 1640 medium. The responder human PBMCs were cocultivated with the stimulator cells, ratio responder/stimulator of 8/3, in complete RPMI 1640 culture medium for 6 days at 37 °C and 5% CO_2_. DNA synthesis of the responder cells was assayed by the addition of 10 *μ*Ci ^3^H thymidine (Perkin Elmer, Zaventem, Belgium) during the last 18 hours of culture. The cells were harvested on glass filter paper (Perkin Elmer, Zaventem, Belgium). After drying, radioactivity was counted in a scintillation counter (TopCount, Perkin Elmer, Zaventem, Belgium).

### 2.3. Generation of Mice with B Cell-Specific Deletion of the ILK Gene

We generated mice with B cell-specific deletion of ILK by means of Cre/LoxP system, a technology to induce site-specific recombination events in genomic DNA. Transgenic ILK^flox/flox^ C57BL/6 mice and CD19^Cre/Cre^ C57BL/6 mice were kindly provided, respectively, by Prof. Dr. Christa Maes (KU Leuven, Belgium) and Prof. Dr. Jan Cools (KU Leuven, Belgium). We used ILK^flox/flox^ C57BL/6 mice, created by the research group of Prof. Dr. René St.-Arnaud (McGill University and Shriners Hospital for Children, Montreal, Canada), wherein LoxP sites were inserted downstream from exons 4 and 12 at the ILK locus. To delete ILK *in vivo* in B cells, ILK^flox/flox^ C57BL/6 mice were bred with CD19^Cre/Cre^ C57BL/6 mice, wherein the expression of Cre recombinase enzyme is placed under the direction of the CD19 promotor, to create CD19^Cre/WT^/ILK^flox/flox^ mice. Animal care was in accordance with the guidelines for laboratory animals from the KU Leuven, and all experiments were approved by the Ethical Committee for Animal Science of KU Leuven (ethical committee, P075-2010 and P107-2015).

**Figure 1:**
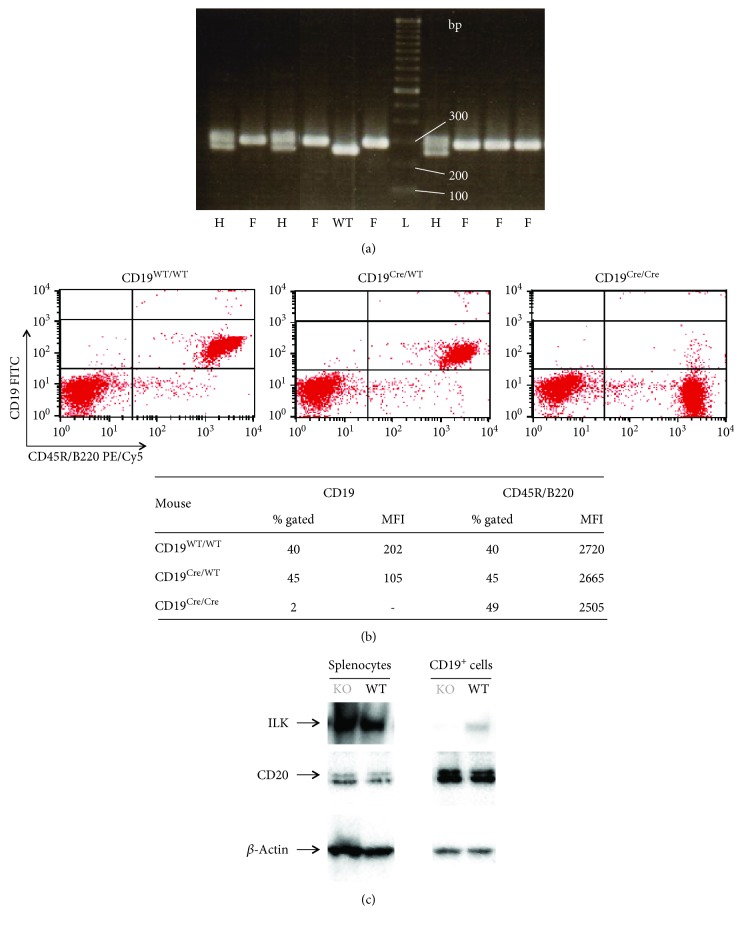
Identification of mice with B cell-specific deletion of ILK. Mice with B cell-specific deletion of ILK were identified through PCR (a) and flow cytometry (b) and Western blot (c). (a) DNA from the tail was amplified by PCR and then separated and visualized on 2% agarose gel to identify the mice with floxed ILK genes: L: TrackIt™ 100 bp DNA ladder; WT: ILK^WT/WT^; H: ILK^flox/WT^; and F: ILK^flox/flox^. (b) Identification of mice with CD19^WT/WT^, CD19^Cre/WT^, or CD19^Cre/Cre^ genotype was done by flow cytometric analysis on the blood that was double-stained with CD19 (FITC) and CD45R/B220 (PE/Cy5). Mice with CD19^Cre/Cre^ genotype did not display CD19 on the outer surface of the B cells. CD19^Cre/WT^ mice still expressed CD19 on the outer surface of the B cells, but at a lower density than CD19^WT/WT^ mice which consequently translated in a lower MFI compared to CD19^WT/WT^ mice. The similar percentages of gated CD45R/B220^+^ cells with equal MFI demonstrated that there was no loss in the B cell population in CD19^Cre/WT^ mice and CD19^Cre/Cre^ mice compared to CD19^WT/WT^ mice. (c) Western blot was performed on splenocytes and on splenic B cells from mice identified as being ILK wild type (WT, CD19^WT/WT^/ILK^flox/flox^) or ILK knockout (KO, CD19^Cre/WT^/ILK^flox/flox^). High expression of ILK was observed in the splenocytes of WT and KO mice, but ILK was not detected in the B lymphocytes of the latter.

Screening of DNA obtained from murine tail for the inheritance of the floxed ILK gene was performed by polymerase chain reaction (PCR) using the following primers: ILK Jax sense (primer sequence 5′ GACCAGGTGGCAGAGGTAAG 3′; Sigma-Aldrich, Diegem, Belgium) and ILK Jax anti-sense (primer sequence 5′ GCTTTGTCCACAGGCATCTC 3′; Sigma-Aldrich, Diegem, Belgium). The final concentrations of the components in the PCR mix for floxed ILK were as follows: 25 U/ml GoTaq® G2 Flexi DNA polymerase (Promega), 1x Green GoTaq® Flexi buffer (Promega), 2 mM MgCl_2_, 200 *μ*M dNTPs, 0.4 *μ*M oligonucleotide primers, and 1 *μ*l of DNA sample for total volume of 25 *μ*l. DNAs were amplified for 35 cycles (95°C for 30 seconds, 58°C for 30 seconds, and 72°C for 40 seconds) in GeneAmp® PCR System 9700 (Applied Biosystems). The size of the WT ILK allele was 245 base pair (bp) and of the floxed ILK allele was 270 bp (Figure 1(a)). Inheritance of the CD19-Cre transgene was determined by SYBR Green dye-based real-time PCR (RT-PCR) using the following primers: CD19-Cre sense (primer sequence 5′ GCGGTCTGGCAGTAAAAACTATC 3′; Sigma-Aldrich, Diegem, Belgium) and CD19-Cre anti-sense (primer sequence 5′ GTGAAACAGCATTGCTGTCACTT 3′; Sigma-Aldrich, Diegem, Belgium). The final concentrations of the components in the PCR mix for CD19-Cre were as follows: 1x SYBR® Green PCR master mix (Applied Biosystems, Halle, Belgium), 0.8 *μ*M oligonucleotide primers, and 1 *μ*l of DNA sample for total volume of 20 *μ*l. DNAs were amplified with the same program as for the identification of floxed ILK allele(s) in StepOnePlus RT-PCR System (Applied Biosystems, Halle, Belgium). A band of 100 bp indicated the mice with a CD19-Cre allele. Distinction between the homozygous and the heterozygous CD19-Cre mice was done by flow cytometry on murine peripheral blood (Figure 1(b)). Murine blood cells were stained with FITC-labelled anti-murine CD19 (Biolegend, ImTec Diagnostics N.V., Antwerp, Belgium) and PE/Cy5-labelled anti-murine CD45R/B220 (Biolegend, ImTec Diagnostics N.V., Antwerp, Belgium) and analyzed with the 3-color Becton Dickinson FACSCalibur apparatus. CD45R/B220, commonly used as a pan-B cell marker, was included in the double staining to allow visualization of B cells in the blood of mice with lowered (heterozygous CD19-Cre genotype) or completely deleted (homozygous CD19-Cre genotype) expression of CD19. Using both PCR and flow cytometry, we were able to select the mice of interest for our investigations: ILK wild type (WT) mice CD19^WT/WT^/ILK^flox/flox^ and ILK knockout (KO) mice CD19^Cre/WT^/ILK^flox/flox^. We used in our studies mice with CD19^Cre/WT^/ILK^flox/flox^ genotype with a partial deletion of CD19 as our ILK KO mice rather than the ones with CD19^Cre/Cre^/ILK^flox/flox^ genotype with a complete deletion of CD19, because, as reported, complete abrogation in expression of CD19 in mice causes defective late B cell differentiation and decreased Ig responses [11, 12].

### 2.4. Protein Detection and Western Blot Analysis

B cell-specific deletion of ILK in CD19^Cre/WT^/ILK^flox/flox^ mice was confirmed by Western blot analysis (Figure 1(c)). Murine splenocytes and highly purified B lymphocytes were lysed, and proteins were separated on NuPAGE® Novex® 4–12% Bis-Tris gel (Invitrogen™, Thermo Fisher Scientific, Niederlebert, Germany) and electrotransferred onto polyvinylidene fluoride membrane (Invitrogen™, Thermo Fisher Scientific, Niederlebert, Germany) and incubated with specific antibodies. Immunoreactive proteins were detected by Bio-Rad Imager (Bio-Rad Laboratories N.V., Temse, Belgium) and normalized to the *β*-actin content. The used antibodies were monoclonal rabbit anti-ILK (4G9; 1/1000; Cell Signaling Technology®, Bioké, Leiden, The Netherlands), monoclonal rat anti-CD20 (AISB12; 1/500; Affymetrix®, eBioscience®, Vienna, Austria), anti-*β*-actin (I-19; 1/1000, Santa Cruz Biotechnology, Heidelberg, Germany), and polyclonal (goat anti-rabbit, rabbit anti-goat, and rabbit anti-rat) Ig/HRP (Dako, Heverlee, Belgium).

### 2.5. TNP-BSA *In Vivo* Assay

Mice were stimulated with T cell-dependent antigen TNP-BSA, a 2,4,6-trinitrophenyl hapten conjugated with bovine serum albumin (50 *μ*g per mice), solved in complete Freund's adjuvant (Sigma-Aldrich, Diegem, Belgium) by subcutaneous injection. The blood was collected by eye puncture before stimulation and on days 7, 10, 14, 21, and 28 poststimulation. On day 28, the mice underwent for a second time stimulation with TNP-BSA and blood samples were taken on days 35 and 42. Analysis of anti-TNP IgM and IgG in the collected sera was done by ELISA procedure in prepared 96-well ELISA plates (coated overnight at 4°C with 5 *μ*g/ml TNP-BSA in phosphate-buffered saline (PBS)) with sheep anti-mouse IgG (H/L) : horse radish peroxidase (HRP) and with goat anti-mouse IgM : HRP (Bio-Rad AbD Serotec, Oxford, United Kingdom), respectively. Peroxidase activity was detected by adding 3,3′,5,5′-tetramethylbenzidine liquid substrate (Sigma-Aldrich, Diegem, Belgium). Reaction was stopped by adding 1 M HCl. Optical absorbance was measured by the ELISA reader at 450 nM.

### 2.6. TNP-Ficoll *In Vivo* Assay

Mice were immunized intraperitoneally with T cell-independent antigen TNP-Ficoll, a 2,4,6-trinitrophenyl hapten conjugated with Ficoll (25 *μ*g per mice), solved in PBS. The blood was collected by eye puncture before stimulation and on days 5, 7, 10, 14, and 21 poststimulation. Analysis of anti-TNP IgM in the collected sera was done by ELISA procedure in prepared 96-well ELISA plates (coated overnight at 4 °C with 5 *μ*g/ml TNP-Ficoll in PBS), as described above.

### 2.7. *In Vivo* Xenoantibody Assay with BHK-570 Cells

Mice were stimulated with intraperitoneal injection of baby hamster kidney (BHK)-570 cells (LGC Standard, ATCC®, Molsheim, France; 5.10^6^ cells in RPMI-medium, 200 *μ*l per mice), and the production of IgM and IgG against the hamster cells was analyzed on days 3, 5, 7, and 10 postimmunization. For analysis of the IgM and IgG titer in the murine sera, 5.10^5^ BHK-570 cells were dispatched per well in a 96-well V-bottom plate and 5 *μ*l of serum sample was added. After incubation of 30 minutes on ice, BHK-570 cells were washed two times with PBS. Secondary antibody (goat anti-mouse IgM antibody-PE or goat anti-mouse IgG antibody-FITC, Biolegend, ImTec Diagnostics N.V. Antwerp, Belgium) was added and incubated for 30 minutes on ice and protected from light. After 3 washes with PBS, IgM and IgG were assessed by flow cytometry with the 3-color Becton Dickinson FACSCalibur apparatus. Ig titer is expressed as mean fluorescence intensity (MFI).

### 2.8. *Ex Vivo* Murine B Cell Activation Assays

Murine splenic B cells were activated by 5 *μ*g/ml lipopolysaccharide (LPS), 0.3 *μ*M ODN1826, or 1 *μ*M resiquimod. After 2 days of incubation, supernatant was taken for analysis of IL6 and TNF*α* with AlphaLISA murine IL6 and TNF*α* kits according to the manufacturer's instructions (Perkin Elmer, Zaventem, Belgium). The proliferation rate of the murine B cells was measured 3 days of poststimulation by adding 10 *μ*Ci ^3^H thymidine (Perkin Elmer, Zaventem, Belgium) in the wells for the last 18 hours of incubation. The cells were harvested on glass filter paper (Perkin Elmer, Zaventem, Belgium). After drying, radioactivity was counted in a scintillation counter (TopCount, Perkin Elmer, Zaventem, Belgium). After 7 days of incubation, supernatant was taken for analysis of IgM and IgG with AlphaLISA murine IgM and IgG kits according to the manufacturer's instructions (Perkin Elmer, Zaventem, Belgium).

### 2.9. Statistical Analysis

Statistical analyses for different experiments were performed using GraphPad Prism 5.00 for Windows (GraphPad Software, San Diego, California, USA). Data are plotted as mean with SEM. The *p* values were calculated by two-tailed Student's *t*-test. *p* values less than 0.05 are considered as significant.

## 3. Results

### 3.1. OSU-T315 Inhibits *In Vitro* Human and Murine B Cell Activation

**Figure 2:**
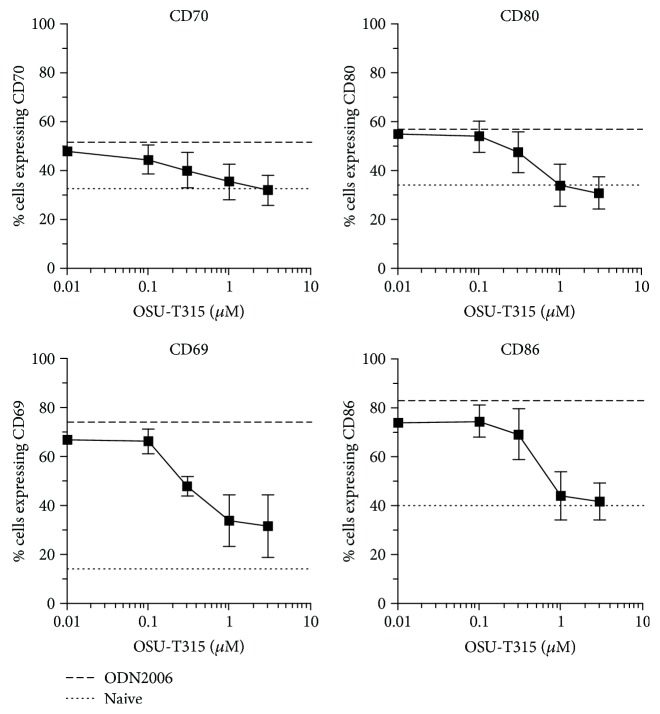
Effect of OSU-T315 on activation and costimulatory cell surface markers after TLR9-mediated stimulation of human B cells. Purified human primary B cells were stimulated with 0.1 *μ*M ODN2006 in the presence of different concentrations of OSU-T315 and were analyzed 24 hours later by flow cytometry. Plotted data are the means with SEM of 3 independently performed experiments.

**Figure 3:**
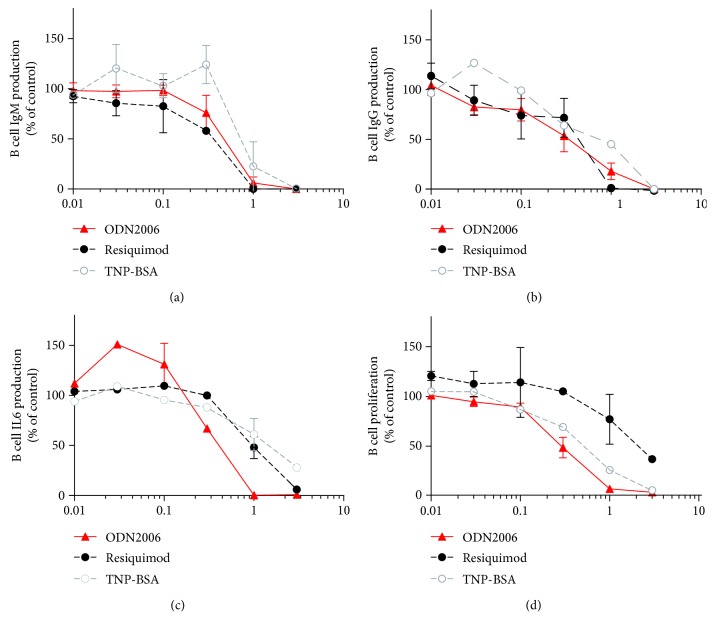
Effect of OSU-T315 on antibody and IL6 production and on human B cell proliferation. Highly purified human primary B cells were activated by different stimulators (0.1 *μ*M ODN2006, 1 *μ*M resiquimod, or 91 *μ*g/ml TNP-BSA). Analysis of IgM (a) and IgG (b) was performed after 7 days of incubation, IL6 (c) was analyzed after 2 days of incubation, and proliferation (d) was measured by ^3^H thymidine incorporation after 3 days of incubation. Plotted data are the means with SEM of 3 independently performed experiments.

A collection of chemical agents was screened in our B cell activation assay using ODN2006-stimulated Namalwa cells [1]. Compound OSU-T315 demonstrated potent inhibitory activity on the upregulation of cell surface markers CD40, CD69, CD70, CD80, CD83, and CD86 on Namalwa cells without affecting viability as measured by WST-1 (Table 1). OSU-T315 was also able to suppress in a dose-dependent manner the upregulation of cell surface markers CD69, CD70, CD80, and CD86 on ODN2006-stimulated human primary B cells with an IC_50_ around 0.5 *μ*M for each marker (Figure 2). In addition, the production of IgM, IgG, and IL6 by purified human B cells in response to ODN2006 stimulation was significantly decreased by OSU-T315 (Figure 3(a–c)). OSU-T315 inhibited also the *in vitro* proliferation of activated B cells with comparable efficacy (Figure 3(d)). The average IC_50_ value in the IgG assay was 0.2 *μ*M, and in the IgM, IL6, and proliferation assays, the average IC_50_ values were 0.5 *μ*M. The inhibition of B cells' activation by OSU-T315 is not restricted to Toll-like receptor (TLR) 9 pathway since B cell activation marker induction, IgM, IgG, and IL6 secretion were also impaired when B cells were stimulated by resiquimod (average IC_50_: CD69 = 1.5 *μ*M, CD70 = 0.6 *μ*M, CD80 = 0.8 *μ*M, and CD86 = 0.9 *μ*M; IgM = 0.4 *μ*M, IgG = 0.3 *μ*M, and IL6 = 1 *μ*M; Figure 3(a–c)). OSU-T315 displayed less potency in the B cell proliferation assay (Figure 3(d)), probably because resiquimod is a relatively moderate stimulator of B cell proliferation. OSU-T315 inhibited also TNP-BSA-triggered B cell proliferation (Figure 3(d)) with an IC_50_ value of 0.6 *μ*M. Inhibitory effect by OSU-T315 was also noticed in the TNP-BSA-induced IgM, IgG, and IL6 production with IC_50_ values of 1 *μ*M, 1 *μ*M, and 1.5 *μ*M, respectively (Figure 3(a–c)). TNP-BSA was not able to induce a significant upregulation of the investigated cell surface markers. In the one-way mixed MLR assay, that evaluates the proliferation of human PBMCs, OSU-T315 proved to be much less effective (average IC_50_ = 3.5 *μ*M) compared to its potency to inhibit the proliferation of human B cells under different stimulatory conditions. This indicates that the inhibitory activity of OSU-T315 is more specific for B lymphocytes as the responder cells in the MLR assay are essentially T cells. OSU-T315 was also active on murine primary B cells as the production of IgM and IgG after stimulation with ODN1826, resiquimod, or LPS was suppressed at comparable potency as in human (average IC_50_ for the three stimuli: IgM = 0.2 *μ*M, IgG = 0.2 *μ*M). OSU-T315 inhibited as well LPS-triggered proliferation (average IC_50_ = 0.2 *μ*M) and LPS-induced production of IL6 (average IC_50_ = 0.1 *μ*M) and TNF*α* (average IC_50_ = 0.5 *μ*M). The obtained data from the phenotypic *in vitro* assays show that OSU-T315 has potential as a B cell inhibitor.

### 3.2. ILK Is Not a Key Kinase in B Cell Activation Pathway

**Figure 4:**
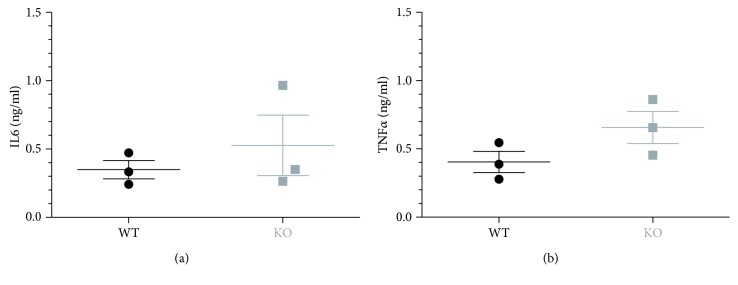
ODN1826 *in vivo* assay on mice with B cell-specific ILK deletion. The production of IL6 (a) and TNF*α* (b) was measured two hours poststimulation in the plasma of ILK WT mice (black, 3 mice) and ILK KO mice (grey, 3 mice).

**Figure 5:**
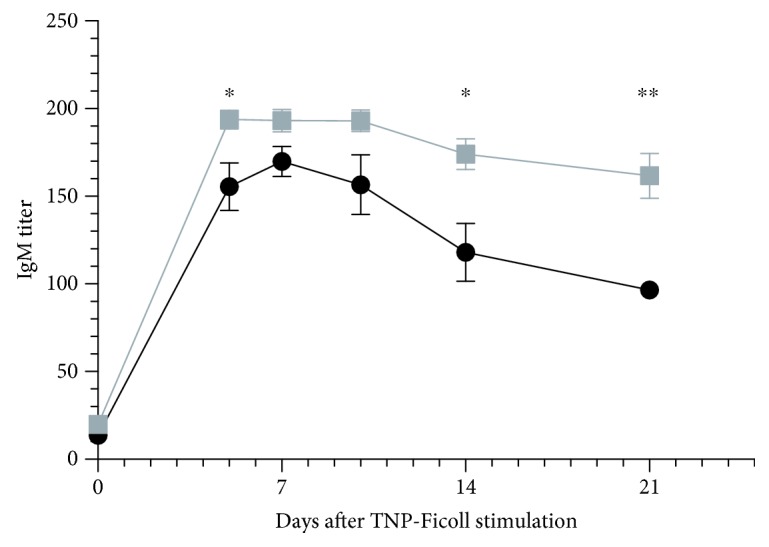
IgM production after TNP-Ficoll immunization in mice with B cell-specific ILK deletion. Anti-TNP IgM production was measured by ELISA over time in the sera of ILK WT mice (black, 6 mice) and ILK KO mice (grey, 6 mice). Plotted data are the means with SEM from one representative of two independently performed experiments (^∗^*p* < 0.05; ^∗∗^*p* < 0.01).

**Figure 6:**
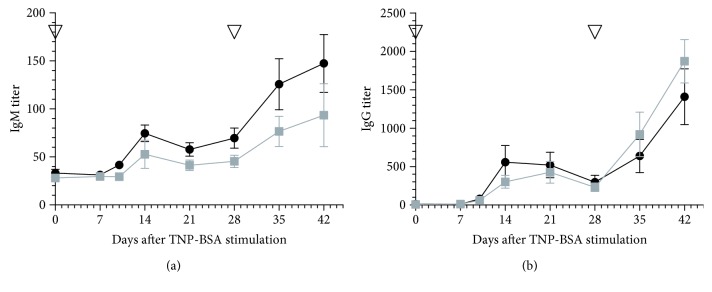
Antibody production after TNP-BSA immunization in mice with B cell-specific ILK deletion. The level of anti-TNP IgM (a) and IgG (b) was measured by ELISA over time in the sera of ILK WT mice (black, 8 mice) and ILK KO mice (grey, 8 mice). TNP-BSA challenges are indicated with reversed triangles. Plotted data are the means with SEM from one representative of two independently performed experiments.

**Figure 7:**
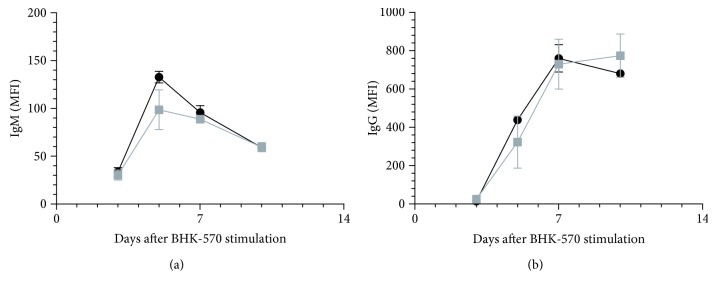
Xenoantibody production against BHK-570 cells in mice with B cell-specific ILK deletion. IgM (a) and IgG (b) production was measured by flow cytometry in the sera of ILK WT mice (black, 3 mice) and ILK KO mice (grey, 3 mice) after immunization with BHK-570 cells. Plotted data are the means with SEM.

**Figure 8:**
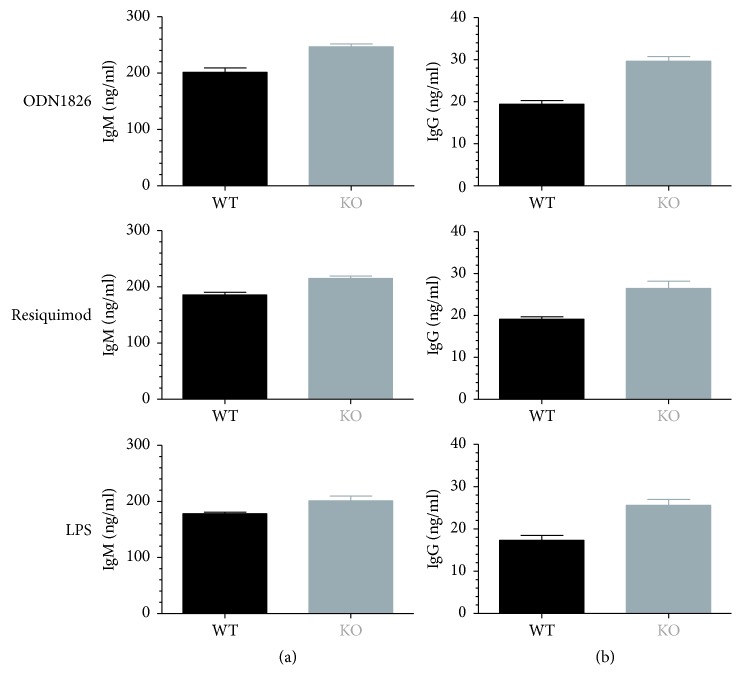
*Ex vivo* antibody production by murine splenic WT or ILK KO B lymphocytes upon activation by different stimuli. Freshly isolated splenic B cells from an ILK WT (black) mouse and an ILK KO (grey) mouse underwent stimulation with ODN1826 (0.3 *μ*M), resiquimod (1 *μ*M), or LPS (5 *μ*g/ml), and production of IgM (a) and IgG (b) was measured after 7 days. Unstimulated B cells from ILK WT and ILK KO mouse produce by average 30 ng/ml IgM and 9 ng/ml IgG. Plotted data are the means with SEM of sextuplets. The graphics show data from one representative of the two independently performed experiments.

**Figure 9:**
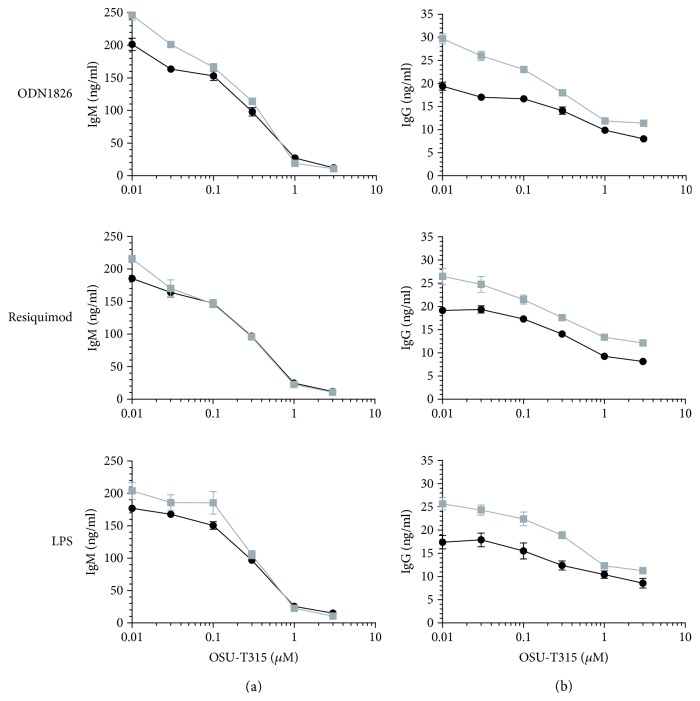
Inhibitory activity of OSU-T315 on Ig production by splenic WT or ILK KO B lymphocytes upon activation by different stimuli. Freshly isolated splenic B cells from an ILK WT (black) mouse and an ILK KO (grey) mouse underwent stimulation with ODN1826 (0.3 *μ*M), resiquimod (1 *μ*M), or LPS (5 *μ*g/ml), and production of IgM (a) and IgG (b) was measured after 7 days. Plotted data are the means with SEM of sextuplets. The graphics show data from one representative of the two independently performed experiments.

**Figure 10:**
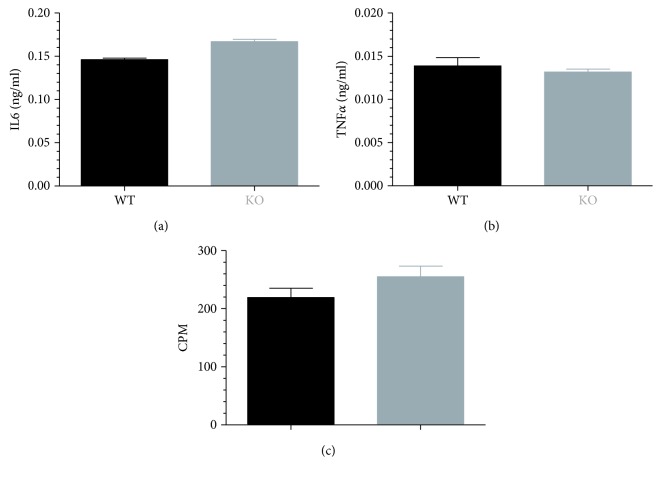
*Ex vivo* IL6 and TNF*α* production and proliferation of murine splenic WT or ILK KO B lymphocytes stimulated by LPS. Freshly isolated splenic B cells from an ILK WT (black) mouse and an ILK KO (grey) mouse underwent stimulation with LPS (5 *μ*g/ml). The production of IL6 (a) and TNF*α* (b) was measured after 2 days and the proliferation (c) after 3 days. Unstimulated B cells from ILK WT and ILK KO mouse produce by average 0.005 ng/ml IL6 and 0.002 ng/ml TNF*α* and have a CPM value of 75. Plotted data are the means with SEM of sextuplets. The graphics show data from one representative of the three independently performed experiments.

**Figure 11:**
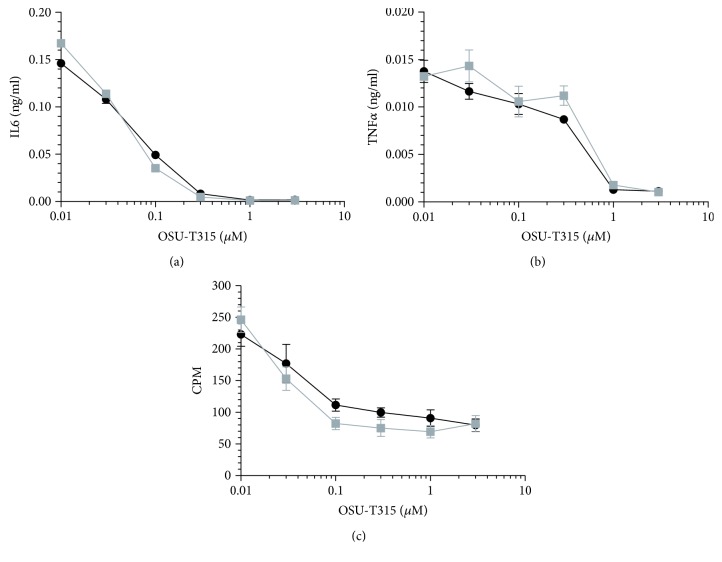
Inhibitory activity of OSU-T315 on cytokine production and proliferation of splenic WT or ILK KO B lymphocytes stimulated by LPS. Freshly isolated splenic B cells from an ILK WT (black) mouse and an ILK KO (grey) mouse underwent stimulation with LPS (5 *μ*g/ml). The production of IL6 (a) and TNF*α* (b) was measured after 2 days and the proliferation (c) after 3 days. Plotted data are the means with SEM of sextuplets. The graphics show data from one representative of the two independently performed experiments.

OSU-T315 is reported as an ILK inhibitor with an IC_50_ of 0.6 *μ*M in an *in vitro* radiometric kinase assay [2]. Our preliminary results with OSU-T315 suggest that ILK could be a promising target in the modulation of B cell activity. In previous studies, some murine models with targeted deletion of ILK have been created to tackle the role of ILK in the different cell populations, including T cells. To our knowledge, ILK has not yet been studied for its role in B cell biology. We generated mice with B cell-specific genetic deletion of ILK to investigate whether the suppressive effect of OSU-T315 on human and murine primary B cells is mediated by ILK and to validate the involvement of ILK in B cell activation pathways. First, we evaluated the effect of ILK deletion on murine B cells *in vivo*. Wild type (WT) mice and knockout (KO) mice with B cell-specific deletion of ILK were stimulated with ODN1826, and plasmatic levels of IL6 and TNF*α* were compared. Surprisingly, there was no significant difference in the level of IL6 and TNF*α* between ILK WT mice and ILK KO mice (Figure 4). Unexpectedly, IgM production was even higher in ILK KO mice compared to ILK WT mice after immunization with T-independent antigen TNP-Ficoll (Figure 5). Similarly, the production of anti-TNP IgM and IgG after stimulation with TNP-BSA, a T-dependent antigen, was not altered in ILK KO mice (Figure 6). Finally, we examined if the B cell-specific deletion of ILK might affect the *in vivo* IgM and IgG production against xenogeneic BHK-570 cells. In this case too, IgM and IgG production was not significantly different between ILK KO mice and ILK WT mice (Figure 7). To clarify the discrepancy between the genetic and pharmacological inhibition of ILK, OSU-T315 was assessed on murine ILK KO B cells in *ex vivo* models. Here again, the B cell-specific deletion of ILK did not impair the antibody production after ODN1826, resiquimod, or LPS stimulation (Figure 8), but, remarkably, OSU-T315 was as efficient in ILK KO B cells as in ILK WT B cells (Table 2 and Figure 9). The production of cytokines and the proliferation (Figure 10) were not substantially altered by a deleted expression of ILK in the B lymphocytes after LPS challenge, but, again, OSU-T315 remained active on ILK KO B cells (Table 2 and Figure 11).

## 4. Discussion

B cells play a central role in the development of cancer [13], autoimmune disorders [14–16], transplant rejection [17–21], and graft versus host disease [22–24] and therefore represent an attractive immune cell population for the development of novel immunosuppressant agents. Using our validated *in vitro* B cell activation assay [1] and various *in vitro* tests, we demonstrated that OSU-T315 influences human B cell function. The weak effect of OSU-T315 in human MLR suggests a rather B cell-specific mode of action.

OSU-T315 is described as ILK inhibitor (IC_50_ is 600 nM in an *in vitro* radiometric kinase assay [2]) modelled from the scaffold that docks into AKT-binding site, but was originally designed to specifically disrupt the interaction of AKT with its binding site on ILK [25]. AKT needs to be phosphorylated on Thr-308 and on Ser-473 for its complete activation. In literature, Western blot analysis on the phosphorylation of AKT on Ser-473 versus Thr-308 in PC-3 cells, a human prostate cancer cell line, and in MDA-MB-231 cells, a human breast cancer cell line, demonstrated that OSU-T315 selectively suppressed AKT phosphorylation on Ser-473, not on Thr-308, in a dose-dependent manner and this was accompanied by parallel decreases in the phosphorylation levels of GSK3*β* and MLC, two downstream targets of ILK [2]. Together, the findings suggested that OSU-T315 might mediate Ser-473 AKT dephosphorylation through inhibition of ILK or through interaction with ILK.

Other small molecules have been explored for their inhibitory activity against ILK, such as KP-392 [26] (also called KP-SD-1), a first generation inhibitor, QLT0267 [27, 28], a second generation inhibitor, and QLT0254 [29], a derivate of QLT0267 (Quadralogic Technologies Inc., Vancouver, Canada). QLT0267 is characterized as a potent ILK antagonist that inhibits the kinase activity of ILK with IC_50_ of 26 nM (IC_50_ QLT0254 is 300 nM) in a cell-free assay using highly purified recombinant ILK [29, 30]. QLT0267 seemed very specific when assessed in a panel of cell-free recombinant kinase assays against 150 protein kinases [27, 29]. However, some conflicting observations have raised doubt about QLT0267's ILK selectivity. Immortalized murine ILK^−/−^ and ILK^fl/fl^ fibroblasts, cultured under 2D or 3D cell culture conditions, were exposed to QLT0267 and radiation [31]. A significantly lower basal cell survival of ILK^−/−^ fibroblasts demonstrated a higher susceptibility of these ILK-deficient fibroblasts to QLT0267 compared to ILK-expressing ILK^fl/fl^ fibroblasts. Radiosensitivity of both ILK^−/−^ fibroblasts and ILK^fl/fl^ fibroblasts was equally enhanced by QLT0267. Moreover, QLT0267 did not reverse ILK-mediated radiosensitization of mutant FaDu cells (a human epithelial cell line from a squamous cell carcinoma of the hypopharynx) that were transfected with a constitutively active form of ILK [31]. These findings give a strong indication of possible extra target of compound QLT0267, and further investigations are warranted.

ILK is an evolutionarily conserved and ubiquitously expressed protein and is assumed to play an essential role in the cell-matrix interactions and in the regulation of cellular processes such as growth, proliferation, survival, differentiation, migration, invasion, and angiogenesis [32]. Increased protein levels and activity of ILK have been associated with the oncogenesis and tumour progression of many types of malignancies (the prostate, ovary, breast, colon, pancreas, stomach, and liver), indicating that ILK represents a potential target for new cancer treatments [2], [33–35]. Whether ILK functions as a protein serine/threonine kinase remains subject of ongoing discussions. The principal argument that ILK is a pseudokinase (thus, functions rather as a scaffold protein and displays no catalytic activity) is based on the primary structure of its unusual protein kinase domain at the C-terminal part, which exhibits similarity to serine/threonine kinases, but lacks specific, highly conserved motifs which are considered to be obligatory for catalytic function [36–38]. It is relevant, but it is not sufficient to conclude that ILK does not function as kinase. After all, functional kinases CASK and VRK3 also have similar deficiencies in canonical residues in their catalytic domains [39]. And several experimental *in vitro* studies have shown that recombinant, highly purified ILK is able to phosphorylate specific substrates such as AKT (on Ser-473) and GSK*β* [37]. On the other hand, murine ILK-deficient fibroblasts [3], chondrocytes [40], or keratinocytes [41] did not show changes in AKT or GSK3*β* phosphorylation, implying that ILK does not function as a kinase in these models or can be replaced by others. In *in vivo* studies with *Caenorhabditis elegans* worms and *Drosophila* flies, it was observed that kinase dead ILK mutants rescued the severe phenotypes caused by ILK deletion in both species, indicating ILK functions as a scaffold protein and not as a kinase in invertebrates [38]. Up to the current time, several discrepancies remain unsolved, especially the issue whether ILK is a bona fide kinase or a pseudokinase. Genetic knockout approaches (with siRNA or with Cre/LoxP method) can deliver further clarification about the relevance and functional role(s) of ILK in the different species, cell types, and biological contexts. A complete deletion of ILK leads to lethality in mice [3], *Xenopus laevis* frogs [42], *Drosophila* flies [43], and *Caenorhabditis elegans* worms [44], emphasizing its importance in embryogenesis and tissue homeostasis. Using the Cre/LoxP recombination system, the mouse ILK-encoding gene has been deleted in several cell types and organs (fibroblasts [3], immortalized macrophages [4], vascular endothelial cells [5], vascular smooth muscle cells [6], chondrocytes [7], heart [8], and mammary epithelial cells [9]). The role of ILK in T cells has been studied using a T cell-specific deletion of ILK, Lck-Cre^+^/ILK^flox/flox^ mice [10]. These mice showed neither gross developmental abnormalities nor thymus defects. Thymocytes from the T cell-specific ILK KO mice were phenotypically indistinguishable from control WT thymocytes, but there was enhanced susceptibility to cell death upon stress conditions like serum deprivation. ILK deletion did not affect *in vitro* T cell proliferation. However, ILK-deficient T cells showed an aberrant response to the chemokines CXCL12 and CCL19, indicating that ILK might have a role in leukocyte chemotaxis and immune cell trafficking [10]. To further characterize the role of ILK in B cell biology, we generated B cell-specific ILK KO mice. In these mice and using various B cell stimulation experiments, the absence of the ILK gene did not reveal a suppressive effect in various B cell phenomena such as ODN1826-induced IL6 or TNF*α* production, TNP-hapten-induced antibody production, and xenoantibody production. When using B cells from these mice *ex vivo*, no alterations were observed either. Moreover, addition of inhibitor OSU-T315 to ILK WT B cells and ILK KO B cells resulted in a very comparable repression in cellular proliferation and production of antibodies, IL6 and TNF*α*. Deletion of ILK protein did not diminish the activity of OSU-T315. Our data indicate that ILK is not critical for murine B cell activation, and that OSU-T315 exerts its B cell inhibitory activity through a mechanism independent of ILK.

In conclusion, we demonstrate that OSU-T315 represents an interesting lead to develop new B cell-specific immunosuppressants for human use even if its precise mechanism of action is not yet fully characterized. Involvement of ILK in these processes is not fully supported by our data obtained in mice that suggest that OSU-T315 has a target besides ILK, and that ILK is not a critical regulator of B cell function in murine B cells.

## Figures and Tables

**Table 1:** *In vitro* phenotypic characterization of OSU-T315 on Namalwa.

*In vitro* analysis	IC_50_ (nM)	SEM (nM)
Flow cytometry		
CD40	270	15
CD69	630	150
CD70	740	55
CD80	240	90
CD83	620	70
CD86	300	95
WST-1	4600	125

Namalwa cells were stimulated with 0.1 *μ*M ODN2006 in the presence of different concentrations of OSU-T315 and were analyzed 24 hours later by flow cytometry. The cytotoxicity of OSU-T315 on Namalwa cells was assessed by the WST-1 assay after 48 hours of incubation of the cells with different concentrations of the compound. Data are the means with SEM of 3 independently performed experiments.

**Table 2:** *In vitro* phenotypic characterization of OSU-T315 on splenic B cells from wild type mice and mice with B cell-specific deletion of ILK.

Stimulus	Readout	Wild type B cells		ILK KO B cells	
		IC_50_ (nM)	SEM (nM)	IC_50_ (nM)	SEM (nM)
ODN1826	IgM	215	21	220	10
	IgG	200	75	135	40

Resiquimod	IgM	235	30	200	20
	IgG	215	65	125	15

LPS	IgM	230	65	210	90
	IgG	235	65	180	100
	IL6	80	20	60	15
	TNF*α*	470	130	690	150
	Proliferation	225	55	105	35

Murine B cells were stimulated with 0.3 *μ*M ODN1826, 1 *μ*M resiquimod, or 5 *μ*g/ml LPS in the presence of different concentrations of OSU-T315. After 7 days of incubation, supernatant was taken for analysis of IgM and IgG. LPS-induced production of IL6 and TNF*α* was measured after 2 days of incubation. The proliferation rate of LPS-activated murine B cells was measured by ^3^H thymidine incorporation after 3 days of incubation. Data are the means with SEM of 2 independently performed experiments.

## Data Availability

The data that support the findings of this study are available from the corresponding author Kristien Van Belle upon reasonable request.

## Abbreviations

BHK: Baby hamster kidney
CD: Cluster of differentiation
DMEM: Dulbecco's modified Eagle's medium
FCS: Foetal calf serum
Ig: Immunoglobulin
IL: Interleukin
ILK: Integrin-linked kinase
KO: Knockout
LPS: Lipopolysaccharide
MFI: Mean fluorescence intensity
MLR: Mixed lymphocyte reaction
ODN: Oligodeoxynucleotide
PBMC: Peripheral blood mononuclear cell
PBS: Phosphate-buffered saline
PCR: Polymerase chain reaction
TLR: Toll-like receptor
TNP: 2,4,6-Trinitrophenyl hapten
WT: Wild type.
